# Factors in postoperative length of hospital stay among surgical patients in a rural Ethiopian hospital: an observational study

**DOI:** 10.11604/pamj.2024.47.212.36450

**Published:** 2024-04-26

**Authors:** Eyasu Samson Kebede, Safa Abdalla, Bete Demeke, Gary Lee Darmstadt

**Affiliations:** 1 Program of Human Biology, Stanford University, Stanford, California, United States of America,; 2 Department of Pediatrics, Stanford University School of Medicine, Stanford, California, United States of America,; 3 Project Mercy, Yetebon, Ethiopia

**Keywords:** Postoperative length, hospital stay, Ethiopia, surgical site infection, community hospital

## Abstract

**Introduction:**

surgical site infection is associated with longer postoperative hospital stays. We explored factors associated with longer postoperative hospital stays among patients in the surgical ward of a primary rural hospital in Ethiopia, where laboratory facilities for microbiological confirmation of surgical site infections were not available.

**Methods:**

an observational study was performed for patients ≥ 18 years of age who underwent elective or emergency surgery from 22^nd^ June 2017 to 19^th^ July 2018. Data were taken from paper-based medical records and patient interviews. The primary outcome was postoperative length of hospital stay. Data were analyzed by multivariable linear regression using Stata software, version 13.

**Results:**

seventy-five patients were enrolled, sociodemographic data was obtained from 14 of these patients by interview, and 44 patients had complete outcome and covariate data and were included in regression analysis. Median length of preoperative hospital stay was 3.0 (interquartile range 2.0) days. Postoperative length of hospital stay was longer by 3.8 days (95% confidence interval (CI) 1.05-6.55; p=0.008), 4.7 days (95% CI 1.64-7.66; p=0.004), and 5.9 days (95% CI 2.70-9.02; p=0.001), for patients 35-54 years, 55-64 years and the 65+ years respectively, compared to patients who were 18-34 years of age. Patients who received preoperative antibiotics stayed 5.3 days longer (95% CI 1.67-8.87; p=0.005) compared to those who were not given preoperative antibiotics.

**Conclusion:**

age and improper use of preoperative antibiotics compound the risk for postoperative length of stay. Infection prevention protocols, including staff training, and surveillance for surgical site infections are critical for improving hospital outcomes.

## Introduction

Increased postoperative length of hospital stay is associated with increased risk for hospital-acquired infections, including surgical site infection (SSI) [1-4]. Development of a hospital-acquired infection, such as SSI, in many cases in turn extends length of hospital stay for diagnosis and treatment of the infection, and complicates treatment of the original condition for which the patient was hospitalized. In high-income settings, SSIs were associated with an increased duration of hospitalization by 7 to 19.5 days [1], placing a significant burden on hospital-related costs. In the United States [5], similar to Europe [6], SSIs increased the length of postoperative hospital stay by an average of 9.7 days and increased costs by $20,842 per admission and $900 million nationally in 2005.

In low and middle-income countries, SSIs are the highest reported category of nosocomial infection at an estimated rate of 11.2 SSIs per 100 patients [7-9]. In low-resource settings, SSI has been reported to extend postoperative hospital stays up to 21 days [8]. Data from sub-Saharan Africa, especially in rural areas, is scarce on associations between SSIs and postoperative length of stay, including risk factors [10]. In Ethiopia, the incidence of SSIs ranged from 10.9% to 75%, depending on the type of surgery [11,12]. In Northwest Ethiopia, significantly higher rates of postoperative infections occurred among patients who stayed in the hospital longer than 15 days after surgery [11].

Research on the prevalence and risk factors of SSIs and predictors of postoperative length of stay is important because surveillance for SSIs and sharing information with surgeons was shown to drive down rates of SSIs [13,14]. One study found that implementing an SSI surveillance program as well as sharing surgeon-specific rates decreased SSIs by about 35% [15].

It has been recognized in the literature examining costs of hospital-acquired infections that length of hospital stay and hospital-acquired infection are interdependent, sharing some risk factors such as age, surgery, and severity of illness for which the patient was hospitalized [16,17]. In a recent study examining the interdependency of hospital-acquired infection and length of hospital stay, Hassan *et al*. reported that extending the length of stay by one day increased the probability of developing an infection by 1.37 percent, and conversely, that the development of infection increased average length of stay by 9.32 days [18].

In low resource contexts, where microbiology laboratory capabilities to directly identify SSIs are often lacking, the interdependency of hospital-acquired infections and length of hospital stay suggests that an analysis of predictors of length of postoperative stay may be useful for gaining insights into reasons for SSIs and other hospital-acquired infections. Our objective is to identify risk factors for postoperative length of stay for surgical patients in the Glenn C. Olsen Memorial Primary Hospital (GCOMPH) in rural Ethiopia. While we do not know precisely the causal relationship between SSI and postoperative length of hospital stay, we assume that identifying risk factors for longer postoperative stays will provide useful insights into potential strategies for prevention of SSI and associated prolonged postoperative hospitalization and costs.

## Methods

**Setting and study size:** an observational study was conducted in patients who underwent major surgery at GCOMPH, located south of Addis Ababa in a low-resource agricultural community with a population of approximately 70,000 people [19]. Project Mercy, a non-governmental organization, manages the 52-bed hospital, which serves approximately 11,000 patients annually. The hospital is the primary surgical center for the local community and also includes pediatric, internal medicine, obstetrics and gynecology, and radiology services. The hospital´s written standard infection prevention policy for the operating room includes monthly fumigation using 10% formaldehyde; strict cleansing, sterilization, and disinfection of materials and equipment after each procedure; and daily cleaning and disinfection of the doctor´s lounge, surgical waiting area, postoperative recovery room, and surgical wards. Related hospital corridors are cleansed and disinfected weekly. Preoperative antibiotic prophylaxis is administered 30-60 minutes prior to incision. Antibiotic selection is based on the body system (area) to be entered and whether the procedure is clean contaminated, contaminated or dirty (treatment continues in this case). Antibiotic prophylaxis is not administered in clean cases.

**Study design and participants:** from 22^nd^ June 2017 to 19^th^ July 2018, patients ≥ 18 years of age admitted for elective and emergency surgeries involving clean and clean-contaminated wounds were included in this one-year retrospective study [20]. For patients who received an operation before 8^th^ July 2018, their medical records were the primary source of information.

**Variables, data sources/measurement, and bias:** the following information was extracted from the records of all study patients: date of admission, date of discharge, sex, age, diagnosis, procedure, primary surgeon, date of operation, duration of operation, whether the procedure was elective or emergency, completeness of surgical safety checklist and whether preoperative antibiotics were given.

For patients who underwent a procedure between 8^th^ to 19^th^ July 2018, when study investigator ESK was on-site, sociodemographic data were also collected prospectively via a questionnaire. Questions were asked regarding primary language; religion; family structure; type of house; access to water, electricity, television, radio, and cellphone; smoking status, and previous hospitalizations or operations.

Ethical approval for this project was provided by the Stanford Institutional Review Board Protocol #45816. Approval to conduct the study was given by both the president of Project Mercy and the head administrator of the hospital. Informed verbal consent was received from every patient who participated in answering the questionnaire. Verbal informed consent was administered by the investigator, ESK, in the presence of a surgical ward nurse, because approximately 40% of reproductive-age adults (15-49 years) are illiterate (unpublished data) and in the rural study setting, agreements are typically made verbally and not in written form. All information was kept confidential.

**Statistical methods:** given that GCOMPH did not have laboratory capabilities to identify SSIs and that hospital-acquired infections and length of hospital stay are interdependent [16,17], we sought to identify factors associated with length of postoperative hospital stay under the assumption that identifying risk factors for longer postoperative stay will provide insights into approaches for preventing SSIs, prolonged postoperative hospitalization and associated costs. We used analysis of covariance, given the mix of numerical and categorical independent variables associated with the primary outcome of interest (i.e. the dependent variable): length of postoperative hospital stay, measured in days from the date of the operation to the date of discharge from the hospital. The risk measure used does not include log odds or odds ratio or risk ratio because the dependent variable is numerical. The interpretation of the coefficient for the numerical independent variable is the change in mean postoperative length of hospital stay for a unit change in the independent variable independent of all other variables. For each level of categorical independent variables, the interpretation is the difference in mean post-operative length of hospital stay between that level and the reference level independent of all other variables. Statistical significance was determined using alpha<0.05.

**Quantitative variables:** we sought to include all possible independent variables hypothesized or shown previously to be related to length of postoperative stay and hospital-acquired infection [16,17,21-28] and for which data was consistently recorded in the patient medical records. Analysis of covariance was adjusted for the following independent variables: sex (male, female), age (years), use of pre-operative antibiotics (yes or no), duration of surgery in hours, whether the patient had previous surgery, length of preoperative stay in the hospital in days, and type of anesthesia used (general, spinal, both, or unspecified). Cases with complete outcome and covariate information were included in the analysis.

## Results

**Participants:** seventy-five patients were enrolled in the study. Among these, sociodemographic data were collected from 14 subjects, half (n=7) of whom spoke Guragigna as their primary language and nine (64%) of whom were Muslim (Annex 1). Half of the 14 patients had 7-12 people living in their houses. In addition, the majority of these 14 patients did not have electricity in their homes, had dirt floors, access to tap water, and owned a radio.

**Descriptive data:** among the 75 subjects who were enrolled, 43 (57%) were male, the median age was 45.0 (interquartile range (IQR) = 25) years and the median length of preoperative hospital stay was 3.0 (IQR = 2.0) days (Table 1). Fifty-six percent of patients (n=42) had surgery lasting longer than 1 hour, 97% (n=73) had elective surgery, and 60% (n=45) had no previous surgeries. The most common procedures were subtotal thyroidectomy for goiter and transurethral electrovaporization for benign prostatic hyperplasia; together, they comprised 56% of the procedures conducted during the study period. Forty-three percent of patients (n=32) underwent general anesthesia and 4% (n=3) were given preoperative antibiotics.

**Table 1:** profile of patients and their surgical procedures at Glenn C. Olsen Memorial Primary Hospital, Yetebon, Ethiopia, 2017-2018

Variables	Frequency (N = 75)	Percentage
**Sex**		
Male	43	57.3
Female	32	42.7
**Age (years)**		
18-34	18	24.0
35-54	27	36.0
55-64	17	22.7
65+	13	17.3
**Preoperative hospital stay (days)**		
≤7	72	96.0
>7	3	4.0
**Duration of surgery (hours)**		
≤1	42	56.0
>1	25	33.3
Unknown	8	10.7
**Types of surgery**		
Elective	73	97.3
Emergency	1	1.3
Unknown	1	1.3
**Previous surgery**		
Yes	21	28.0
No	45	60.0
Unknown	9	12.0
Head surgeon	75	100.0
**Procedures**		
Subtotal thyroidectomy for goiter	22	29.3
Transurethral electrovaporization for benign prostatic hyperplasia	20	26.7
Excision	8	10.7
Hernia repair	7	9.3
Mastectomy	2	2.7
Hemorrhoidectomy	2	2.7
Other	14	18.7
**Types of anaesthesia**		
General	32	42.7
Spinal	29	38.7
Both	3	4.0
Unspecified	11	14.7
**Preoperative antibiotics**		
Yes	3	4.0
No	49	65.3
Unknown	23	30.7

**Outcome data, main results, and other analyses:** forty-four patients had complete information on the outcome of length of postoperative hospital stay and all covariates and were included in regression analysis. Postoperative hospital stays for patients in the 35-54 year, 55-64 year, and the 65+ year age categories were longer by 3.8 days (95% confidence interval (CI) 1.1-6.6, p=0.008), 4.7 days (95% CI 1.6-7.7, p=0.004), and 5.9 days (95% CI 2.7-9.0, p=0.001), respectively, compared to the 18-34 year age category (Table 2). Postoperative hospital staya for patients who were given pre-operative antibiotics was 5.3 days longer (95% CI 1.7-8.9, p=0.005) compared to those who did not receive preoperative antibiotics. Patients who had a pre-surgery hospital stay of >7 days stayed 4.1 days (95% CI -8.6-0.4, p=0.071) fewer days in the hospital after surgery, and patients who had a previous surgery stayed about 1.8 days (95% CI -0.3-3.9, p=0.091) longer than those who had not undergone a previous surgery.

**Table 2:** analysis of covariance model associated with postoperative hospital stay at Glenn C. Olsen Primary Memorial Hospital, Yetebon, Ethiopia (N=44)

Variables of Interest	Coefficient (95% confidence interval)	P-value
**Sex**		
Male	Reference	
Female	-1.82 (-4.11, 0.47)	0.115
**Age**		
18-34	Reference	
35-54	3.80 (1.05, 6.55)	0.008*
55-64	4.65 (1.64, 7.66)	0.004*
65+	5.86 (2.70, 9.02)	0.001*
**Preoperative hospital stay (in days)**		
≤7	Reference	
>7	-4.13 (-8.63, 0.37)	0.071
**Previous surgery**		
No	Reference	
Yes	1.80 (-0.30, 3.91)	0.091
**Preoperative antibiotics**		
No	Reference	
Yes	5.27 (1.67, 8.87)	0.005*

* The coefficient is statistically significant at the p<0.05 value

None of the other variables of interest, such as sex, type of anesthesia used, duration of surgery, and type of surgery (elective or emergency) were statistically associated with length of hospital stay.

## Discussion

In this study in a rural community hospital in Ethiopia, given known interdependencies between hospital-acquired infection and length of hospital stay and the lack of laboratory capabilities to definitely diagnose surgical infections, we sought to identify risk factors for postoperative length of stay and to gain insights into potential preventative factors for SSIs, for which definitive data is lacking. We found that longer postoperative hospital stay was associated with older age and use of preoperative antibiotics. Previous studies have shown that age is a predictor of postoperative complications [21,22]. Although the protective benefit of preoperative antibiotics is well-established, proper use of antibiotics is essential to maximize their advantage [23,24]. Our results are consistent with some observational studies and may be due to confounding variables such as more frequent use of antibiotics for patients considered at high risk for infection or even medical errors - factors for which we had limited information [25]. Further research is needed on the association of preoperative antibiotics with postoperative length of hospital stay in our setting.

We also found that a preoperative stay of 7 days or more was associated with a shorter postoperative hospital stay by nearly 4 days, although the relationship was of borderline statistical significance (p=0.077). Studies have shown that a shorter length of preoperative hospital stay is associated with a lower risk for SSIs [26-28]. Because surgical patients typically are admitted to GCOMPH during the weekend and their procedures are scheduled throughout the week, preoperative length of stay was not necessarily indicative of how sick the patient was.

Our study can help to stimulate a more targeted approach to preventing and responding to postoperative infections, including SSIs, at GCOMPH and other similar settings. Awareness of the seriousness of post-operative infections in GCOMPH is high; for example, any suspected SSI is treated as an emergency by the head surgeon, and a meeting is called with the nursing staff to discuss its management immediately. However, many nurses are unfamiliar with infection prevention protocols when they begin to work at GCOMPH, merely observing the different steps that are taken, with no systematic training process on infection control and prevention or on documentation of risk factors, treatment, and outcomes.

Implementing staff training in systematic clinical protocols and documentation is critical in improving patient outcomes. Our study draws further attention to those at high risk, making surveillance of the length of stay and SSIs particularly important among the high-risk groups a key next step. This research alongside quality improvement studies can draw further attention to the importance of more structured training on infection prevention, recognition, and treatment protocols.

During the course of this study, we found that although the surgical safety checklist is already being used in the surgical ward of GCOMPH, it is not used for every patient undergoing an operation. Because research has shown that proper use of a surgical safety checklist can lead to decreased inpatient complications such as SSIs and mortality, it is essential to better enforce the use of the checklist [29,30]. More complete information regarding preoperative antibiotics is needed in medical charts, such as information on time, dose, and frequency of antibiotic administration. Lack of consistency when filling out forms within medical charts is due in part to language barriers at GCOMPH - since forms are in English whereas conversation occurs in Amharic - as well as to lack of accountability. These are common issues in rural hospitals and designing a short and simple form for record keeping by hospital workers is advised. This could improve the clarity of medical charts, ease the burden on hospital workers, and simplify the process of surveillance for SSIs.

There is great potential for the success of these measures in preventing SSIs due to the design of GCOMPH. Previous research has shown the relationship between improving surgical ward air hygiene and reduction in cross-infection of wounds [31,32]. The separation of wards at GCOMPH by walkways and a courtyard was intentionally incorporated into the design process in order to enhance air circulation. Concrete masonry units were also used for the floors in order to prevent infection problems such as fungus from collecting. The placement of each door, window, room, and ward was meticulously planned to provide for air movement and cross-ventilation. In the care management center where patients are prepared for operations, there was also a room that was partitioned as an isolated area, designed for the purpose of separating patients if they had an infection develop.

Our study had several limitations. Due to limited capabilities, it was not possible to directly identify SSIs through microbiological testing at GCOMPH. Accurately identifying an SSI clinically based on patient health records also was not possible because no document exists where a physician or a nurse reliably recorded whether a patient had an SSI or not. All patient health records were hard-copy and hand-written; therefore, fewer variables could be analyzed due to missing data, which limited the robustness of the analysis. For example, the presence of a pre-existing wound could influence the choice of preoperative antibiotics; however, the presence or absence of preexisting wounds was not consistently noted in the patient´s medical records. Postoperative stay is limited in its association with SSI as several studies have shown that >50% of SSIs present after discharge [33-35]; however, we lacked systematic information on the follow-up of our patients. Another limitation is the small sample size, given that GCOMPH is a community-based primary hospital. These are some of the realities of working in a rural, low-resource setting. Nevertheless, calling attention to these issues is an important first step in catalyzing actions to improve the prevention of nosocomial infections and to minimize hospital length of stay in hospitals in low-resource settings such as rural Ethiopia.

## Conclusion

Many rural hospitals in low-resource settings lack capabilities for microbiological confirmation of SSIs, and the use of postoperative length of hospital stay may provide insight into risk factors that when managed as part of a package of care may contribute to the reduction in risk for SSI. We found that older age was associated with longer postoperative stay, consistent with prior research. Given existing data, including from East Africa, showing benefits of preoperative antibiotics, our finding of an association between preoperative antibiotics and longer postoperative stay requires further investigation into potential explanatory covariates such as comorbidities, the severity of illness, and details of preoperative patient management which we were unable to explore, given the limitation of our data. It is recommended that a hospital review committee be formed to periodically examine medical charts for information relevant to surveillance, such as data on postoperative length of stay and antibiotic use. In addition to ensuring greater accountability for the staff, a hospital review committee can also supply additional training to workers and provide surveillance and monitoring to control against future infections that may arise within the hospital.

### What is known about this topic

- *Postoperative length of stay is a well-established predictor of surgical site infections;*
- *Prevalence of surgical site infections is well-established in the developed world*.

### What this study adds

- *We provide data on postoperative length of stay in a rural sub-Saharan African hospital and its association with other predictors of surgical site infections; such an analysis from a rural African hospital is rare, given the widespread lack of microbiological capabilities for confirming surgical site infections in these settings, and provides insights into potentially modifiable factors associated with surgical site infections through the use of more readily available data on length of postoperative hospital stay;*
- *Preoperative antibiotic use was significantly associated with longer hospital stays by 5 days; surveillance of the length of stay and for surgical site infections is particularly important among high-risk groups, such as elderly patients;*
- *Staff training in systematic clinical protocols and documentation, and regular hospital review of cases, are critical in improving patient outcomes*.

## Annex

**Annex 1**: sociodemographic data of patients undergoing surgical procedures at Glenn C. Olsen Memorial Primary Hospital, Yetebon, Ethiopia, July 8-19, 2018 (PDF - 336 Kb)
